# The Indigenous Health Research Priorities Study: family research priorities identified by Aboriginal and Torres Strait Islander communities in Queensland, Australia

**DOI:** 10.1186/s12889-026-27521-y

**Published:** 2026-06-08

**Authors:** Kai Wheeler, Salma M Ahmed, Luciana Massi, Loretta Weatherall, Davina Smith, Rhiannon Friday, Emily S Dorey, Bronwyn Fredericks, Maree Toombs, Kym M Rae

**Affiliations:** 1 School of Human Movement and Nutrition Sciences, The University of Queensland, Brisbane, QLD Australia; 2 Indigenous Health Research Group, Mater Research Institute- The University of Queensland, Level 3, Aubigny Place, Brisbane, QLD 4101 Australia; 3 Office of the Deputy Vice Chancellor Indigenous Engagement, ARC Centre for Indigenous Futures, The University of Queensland, Brisbane, QLD Australia; 4 School of Population Health, Faculty of Medicine and Health, University of New South Wales, Sydney, NSW Australia; 5 Faculty of Health Medicine and Behavioural Sciences, The University of Queensland, Brisbane, QLD Australia

**Keywords:** Perinatal, Family health, Community-led research, Participatory Action Research, Indigenous, Social justice, Equity, Yarning, Aboriginal and Torres Strait Islanders

## Abstract

**Background:**

Aboriginal and Torres Strait Islander peoples hold deep cultural strengths, kinship structures, and knowledge systems that are central to health and wellbeing. Yet, health research in Australia has historically been dominated by deficit-based approaches, often overlooking Indigenous knowledges and leadership.

**Methods:**

The Indigenous Health Research Priorities (I-Priorities) study aimed to prioritise Indigenous community voices in identifying health research priorities across Queensland, with a focus on early life, perinatal and family health. Using Indigenous Methodology and a participatory action research framework, communities across Far North, North, Central and South-East Queensland identified and refined their health research priorities. This was achieved in two phases: initial yarning sessions followed by Delphi workshops. Data were analysed thematically and validated through community review processes to ensure cultural integrity.

**Results:**

The study engaged 403 participants through 40 yarning sessions and 13 Delphi workshops conducted between May 2022 and October 2024. The study included participants across diverse age groups, with 80.5% identifying as Indigenous. Communities consistently highlighted key health priorities, with an overwhelming number of participants identifying access to health services, particularly transport, affordability, and overcoming institutional racism. Social and emotional wellbeing, along with family and domestic violence, also emerged as top priorities. Overall, the findings affirm that Indigenous-led research provides solutions grounded in cultural strengths and guided by principles of self-determination.

**Conclusion:**

The I-Priorities study offers a clear roadmap for aligning research, service delivery and policy with priorities defined by Aboriginal and Torres Strait Islander communities, fostering stronger beginnings, healthier families and intergenerational wellbeing across Queensland. Importantly, findings from communities underscore that health equity requires more than biomedical solutions and demands sustained investment in cultural, social and structural determinants of health.

**Supplementary Information:**

The online version contains supplementary material available at 10.1186/s12889-026-27521-y.

## Background

Aboriginal and Torres Strait Islander peoples possess (the term Indigenous will respectfully be used hereafter) enduring cultural strengths, kinship structures, and knowledge systems that have supported health, wellbeing, and resilience across thousands of generations [1, 2]. These community-led practices have sustained children, families, clans and Nations through complex challenges and continue to inform what it means to be well, strong and connected. Increasingly, Indigenous communities and researchers are asserting the importance of health and wellbeing research that reflects this worldview, one that recognises health not solely as the absence of disease, but as an interconnected relationship between people, Country, culture and collective wellbeing [3, 4].

Historically, health research and policy in Australia have disproportionately emphasised deficit-based narratives, particularly when applying frameworks such as the Developmental Origins of Health and Disease (DOHaD). While DOHaD research has provided valuable insights into the long-term impact of early life exposures, [5] it often positions Indigenous peoples through a lens of poor health, vulnerability and risk. This framing can obscure the cultural knowledges, agency, and resilience embedded within communities, reinforcing colonial structures rather than challenging and dismantling them [6]. When reimagined in partnership with Indigenous peoples, DOHaD may offer opportunities to illuminate how intergenerational health and wellbeing are shaped not only by early life exposures but also by protective cultural practices, kinship and historical continuity [7–9]. Guided by Indigenous leadership and culturally safe methodologies, such research can support upstream interventions that honour the role of family, kinship and Country in nurturing healthy pregnancies and promoting the wellbeing of children. Rather than pathologising Indigenous life courses, DOHaD can instead affirm cultural resilience and support self-determined solutions [10].

It should also be noted that in the current article, it would require over-simplification and be inappropriate to attempt to summarise the many ways in which Aboriginal and Torres Strait Islander peoples have been silenced in Queensland and more broadly in Australia. For example, forced removal of children has been a divisive and destructive practice used by colonisers throughout Australia [11], and has silenced many communities, but to discuss here would not hold enough gravitas. In recent examples, silencing has included the Queensland Government repealing the pathway to treaty and the complexities of this are beyond the scope of this paper. The silencing of Aboriginal and Torres Strait Islander peoples has been and still is systematic and ruthless in an attempt to enact genocide on proud peoples. Readers who would like to learn more about the socio-historical contexts of colonisation are referred to Kidd (1997) [12], who provides an in-depth account in Queensland, Australia, for which the current study is set. Despite this, it is clear that empowering the voices of Aboriginal and Torres Strait Islander peoples must be part of a self-determined future and may facilitate a level of healing from the trauma of invasion and occupation by colonisers.

The Indigenous Health Research Priorities (I-Priorities) study responds to the limitations of DOHaD and the socio-historical practices of colonisation by privileging community voices, lived experiences and Indigenous-led design [13]. The I-Priorities study engaged directly with Indigenous communities across Queensland to identify health priorities that matter to families spanning preconception, pregnancy, birthing and early childhood. By situating early life health within the social and cultural determinants of health, the I-Priorities study reframes DOHaD research as culturally responsive, strengths-based and community-led.

Social and cultural determinants of health are central to Indigenous health and wellbeing. These determinants include not only structural conditions, such as housing, education, and access to healthcare, but also cultural identity, kinship and connection to Country, which act as protective factors enabling families and communities to thrive [14, 15]. Despite growing recognition of these determinants in policy, health research and services often fail to engage deeply with cultural dimensions, treating them as peripheral rather than foundational [16]. Aboriginal Community Controlled Health Organisations (ACCHOs) exemplify how health care can be culturally safe, holistic, community-driven and responsive to the needs of Indigenous people [17]. ACCHOs are the governing bodies that operate Aboriginal and Torres Strait Islander Community Controlled Health Services (ACCHSs), which are local, community-controlled, managed, and operated primary health care services [18–21]. Together, they deliver comprehensive care that includes clinical services, health promotion, cultural safety initiatives and strong community engagement [22]. There are currently 148 ACCHOs across Australia, representing a strong and enduring presence in the health system. These organisations are grounded in principles of self-determination and cultural integrity, improving access and outcomes while addressing systemic barriers such as racism [23]. Their proven success makes ACCHSs trusted sites for programs that strengthen Indigenous health and wellbeing and support the shift toward healthier communities [23].

The I-Priorities study embedded Indigenous knowledge systems and leadership into the process of defining research directions. In doing so, it shifts the focus from narrowly defined risk factors to strengths-based, community-driven transformation [24]. This approach ensures that research not only contributes to improved perinatal and early childhood outcomes, but also strengthens cultural continuity, belonging and identity, factors Indigenous communities consistently identify as essential for health and wellbeing [2].

This paper aims to present the findings of the I-Priorities study conducted across Queensland, Australia. The study identified and prioritised health research topics that reflect community-defined needs, with particular attention to perinatal and early childhood health. In doing so, it not only contributes to the evidence base for improving Indigenous child and family health but also demonstrates a model for how health research can uphold Indigenous data sovereignty, cultural integrity and relational accountability. These priorities will inform the co-design of future studies, including the Strong Families Study in Queensland, ensuring that research efforts align with what communities’ value most and that which promotes intergenerational strength.

To achieve the ambitious aim of the current project, we drew from Indigenous Standpoint Theory [25]. This theory was used as a theoretical basis whereby researchers conducting yarning sessions were Indigenous and well-versed in the applications and limitations of Indigenous theories [26]. Great emphasis was placed on that fact that the current research was of benefit to the community and priority was given to community give-back sessions and materials [26]. This operated under Rigney’s Indigenist Research approach emphasising freedom as imperative, political integrity and privileging Indigenous voices [27]. This theoretical basis gave the research team a strong capacity to promote self-determination and build capacity of Community during the research process.

## Methods

The Indigenous Health Research Priorities study engaged communities across various regions in Queensland, Australia, to identify health research priorities for mothers, fathers and babies. Please refer to the published study protocol for more details [13]. Through the current study, we connected and engaged with Indigenous communities to determine health and medical research priorities for young families during preconception, pregnancy, postpartum and early childhood. We mapped these priorities for Indigenous women, men and their young family members across these periods through a collaborative process. Several reports have already been published for the Far North Queensland [28], Darling Downs [29], Townsville [30], Palm Island [31], and Rockhampton regions [32]. The final report for the Woorabinda region is to be published soon.

During the preliminary phase of the study in 2020, face-to-face consultations were conducted with the Queensland Aboriginal and Islander Health Council (QAIHC), the peak body representing ACCHS member organisations across Queensland. With QAIHC’s endorsement and support, health services were identified, and initial introductions were facilitated. Prior to study commencement in 2022, regular meetings were held between researchers and the health services, fostering the development of rapport and trust essential for collaborative engagement. This extended preliminary phase was critical not only for establishing robust planning and processes with health services, but also for navigating the challenges that occurred as waves of the COVID-19 pandemic.

### Study setting

**Fig. 1:**
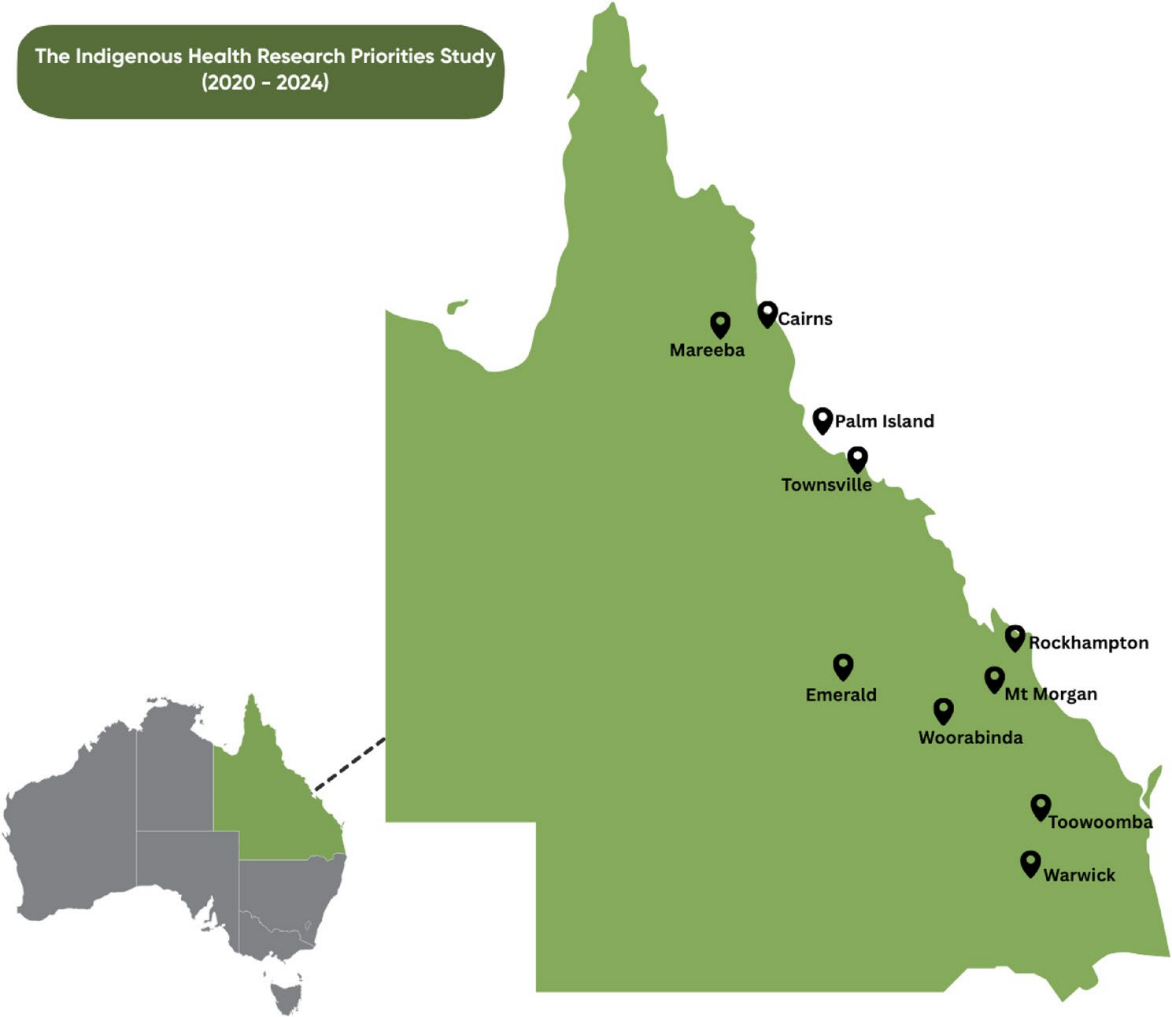
The I-Priorities study sites across Queensland, Australia

Researchers conducted the current study in Queensland, Australia, the only state that includes Torres Strait Islander territories in addition to Aboriginal Country (may be known as territories in some contexts) [33]. Several ACCHS in Queensland partnered in this research. These included in Townsville (Townsville Aboriginal and Islander Health Service (TAIHS)), Palm Island (Palm Island Community Company), Woorabinda (Yoonthalla Services Woorabinda), Rockhampton (Bidgerdii Community Health Service), Cairns (Wuchopperen Health Service and Mookai Rosie Bi-Bayan, Far North Queensland region), Mareeba (Mulungu Aboriginal Corporation Primary Healthcare Service, Far North Queensland region), along with Carbal Aboriginal Medical Services (Darling Downs region) in Warwick and Toowoomba (Fig. 1). At each study site, study champions were identified to liaise with the research team and assist in planning study visits and recruitment.

### Recruitment

Eligible participants included Indigenous community members, with particular emphasis on pregnant women, mothers, fathers, extended family members, kinship carers and Elders. Participation also included health professionals working in maternal and infant health, including those providing care and support from the preconception stage through to early childhood. Their inclusion in the study was consistent with guidance from community-controlled partner organisations. Many of these participants were embedded within their communities and brought perspectives shaped by cultural, family and community, in addition to their professional roles. Also, health professionals included individuals who identified as either Indigenous or non-Indigenous members of the community as they had significant experience in their respective areas of health research needs for their communities. Individuals interested in the study were provided with a Participant Information Consent Form (PICF) in person to familiarise themselves with the study and provide written informed consent prior to the commencement of study activities.

### Study procedure

**Fig. 2:**
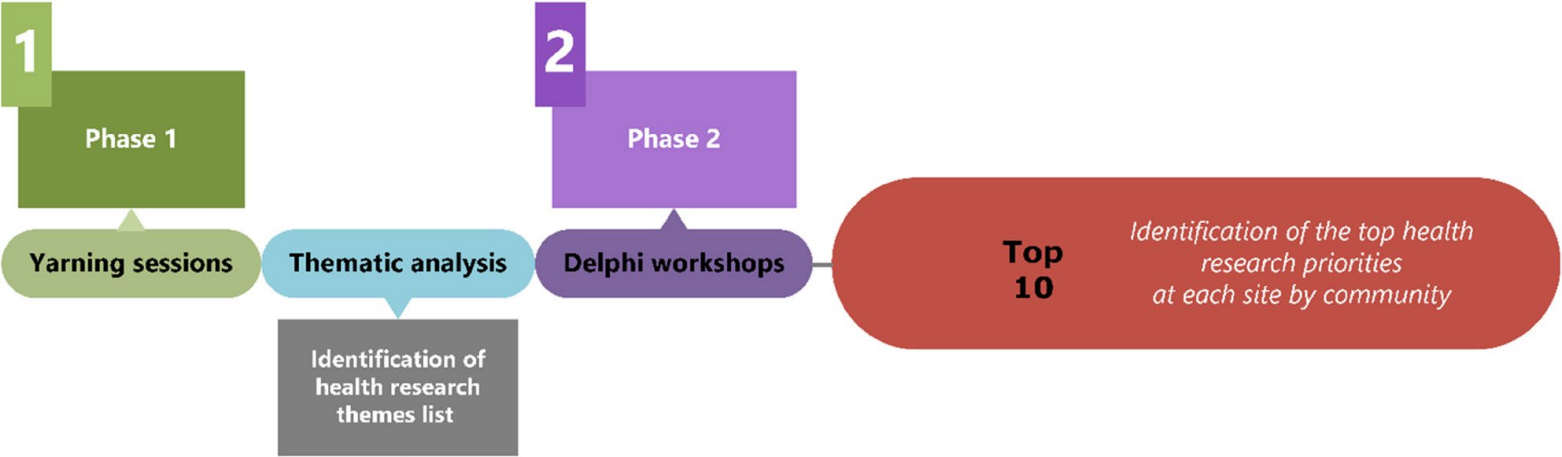
Phases of the I-Priorities study

The current study uses a partnership approach to working with Indigenous communities. This includes the design of the methodology, collection of data and confirmation of results, all through a Participatory Action Research (PAR) framework, which can be effectively utilised with Indigenous populations [34–36]. PAR refers to a collaborative approach that empowers communities most affected to co-lead research and decision-making, ensuring reciprocity and promotion of community wellbeing [37]. This PAR approach is effective in Indigenous populations where relational underpinnings are essential. In this study, PAR was utilised across two phases (Fig. 2).

#### Phase 1: Yarning sessions

Phase 1 of the study involved a series of focus groups conducted using a yarning methodology as reported in the protocol paper for this project [13] and which has been effectively used in a range of other Indigenous-led Indigenous population-based research studies [38–40]. This phase formed the foundational, participatory component of the study. Thus, it should be noted that Phase 1 is not reported in the current paper but its findings form the foundation for Phase 2 as presented here. The yarning approach employed through the project is a proven culturally safe approach to data collection that involves both social and research-focused yarning in an informal and relaxed environment for exploring topics relevant to the study [41]. Central to this methodology is a need for the researcher to build a relationship that was accountable to the research participant and drew out participant stories regarding their lived experiences, feelings, thoughts and ideas on the health priorities for Indigenous families in Queensland [24, 41]. This relationship-building was strengthened through ongoing communication with community-controlled health services during waves of the COVID-19 pandemic through this study, as it was in other studies [38, 42, 43]. These findings demonstrate the importance of ongoing, consistent engagement with communities during difficult times, rather than withdrawal by researchers. Furthermore, pre-existing relationships between the lead researcher (KMR), other researchers involved in the project, and health service managers further contributed to establishing a culturally safe and welcoming research environment [13].

A visual yarning tool was used to prompt participant discussion across different topics [13]. This was complemented by semi-structured questions that could be used by researchers if necessary to facilitate conversation if the yarning drifted off track. Audio recordings were used in this phase to capture the discussions. The research yarning component typically lasted approximately 45 min to an hour and a half per site, with total session length varying depending on group size and participant engagement. Following Phase 1, the main health themes and sub-themes were identified through thematic analysis of the transcripts [44]. During this phase, participants were also asked to complete a brief demographic survey.

#### Phase 2: Delphi workshops

**Fig. 3:**
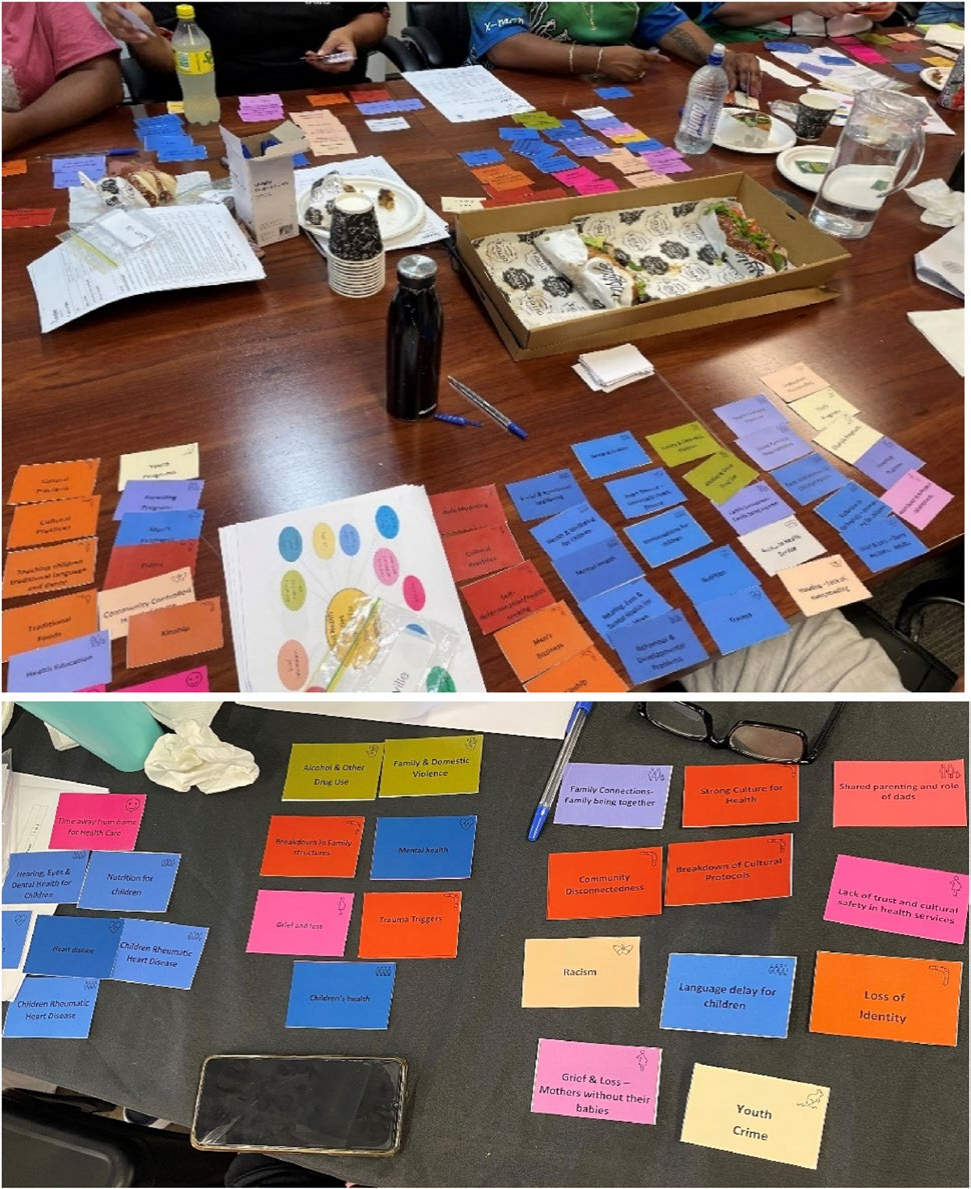
Themed cards in use at an I-Priorities workshop in Queensland, 2024

The research team conducted a separate study visit for Phase 2, which employed the Delphi technique, a PAR method designed to facilitate structured group dialogue to build consensus on the top health priorities in communities through a series of stages [45]. The Delphi technique is not commonly employed with Indigenous communities due to the time required, but it can be effective in gaining a deeper understanding of issues.[46]. For new and returning participants joining in Phase 2, the recruitment and consent processes established in Phase 1 were followed. During the Delphi workshops, participants reviewed the health themes and sub-themes identified from Phase 1. Seated around tables, participants were provided with an individual set of colour-coded theme cards resembling playing cards (Fig. 3).

In Round 1, participants were asked to halve their set by sorting each card into ‘Yes’ (important to me) or ‘No’ (not important to me). The ‘Yes’ votes were tallied up and presented to everyone by a researcher, for example, on a whiteboard. The top two-thirds of these votes were retained for Round 2. The final score of each theme is the sum of the scores of all participants. In total, two to three rounds were conducted during this Phase, resulting in the identification of the top ten health priority areas at each site.

### Data analysis

A detailed description of the Phase 1 and 2 analyses can be found elsewhere [13]. Briefly, transcripts from yarning sessions were reviewed for accuracy before undergoing thematic analysis. Transcripts and audio files were de-identified and were only accessible to the research team. Using open and inductive coding, key themes were identified and refined in consultation with Senior Indigenous researchers. NVivo 12 software (QSR International, Melbourne, 2012) was used to manage and organise the data. The analysis followed a six-step process: data immersion, initial coding, theme identification, theme review, theme definition and report production [44].

To ensure cultural sensitivity and respect for cultural protocols, data collection and analysis related to Women’s and Men’s business were led by female and male identifying Indigenous researchers, respectively. Demographic data was recorded, cleaned and entered into Research Electronic Data Capture (REDCap) [47], a secure, web-based software platform, for further analysis.

#### Ranking of top health themes for the combined sites

To determine the top-ranked health themes for each study site, only themes that were consistently prioritised by more than one health service were included. These commonly agreed themes were then ranked. Each rank was assigned a corresponding point/score. For example, first ranked theme received 10 points, second ranked received 9 points, third ranked received 8 points, and so on. Themes with higher total points were placed higher in the overall ranking, while those with fewer points were ranked lower. This approach allowed the identification of the top ten themes across participating sites in Queensland. Unlike other priority areas, which were identified and ranked as stand‑alone themes, social determinants of health were grouped into a single category for the purposes of ranking to enable comparison across sites.

## Results

The I-Priorities Study was conducted between May 2022 and October 2024 (Supplementary Table 1). During this period, 40 Yarning sessions and 13 Delphi based workshops were held across Queensland, resulting in a total of 403 participants being engaged. It is important to note that some individuals participated in both phases at some sites.

The demographic characteristics of participants in Phase 1 (*n* = 276) and Phase 2 (*n* = 127) of the study are presented in Supplementary Table 2. Across both phases, the sample was predominantly female (Phase 1: 86.1%; Phase 2: 88.2%), with most participants reporting being married or in de facto relationships. The majority of participants identified as Aboriginal only (64.0%), while 6.3% identified as Torres Strait Islander only, and 10.2% identified as both Aboriginal and Torres Strait Islander. Participants were mostly aged between 30 and 59 years, indicating a largely middle-aged cohort. Over half of the participants in both phases had post-secondary education, and employment was high, especially in Phase 2 (74.8%). Most had children (81.3%), with fewer reporting grandchildren (35.8%) or non-biological dependents (16.4%). Annual household income levels varied, though many participants earned between $49,901 and $81,450. The majority of Indigenous participants were active in their communities, often working in health or community organisations.

**Fig. 4:**
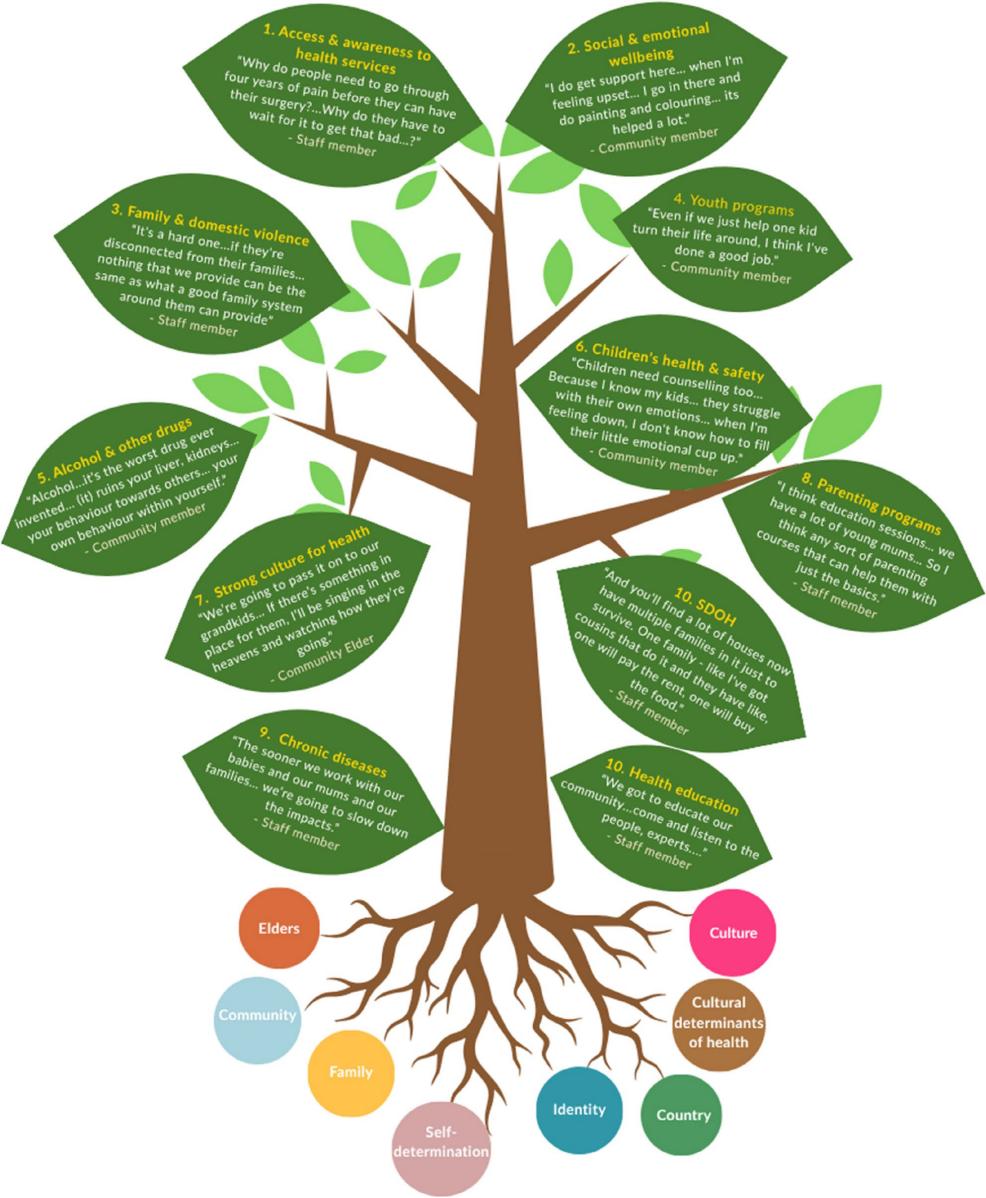
The top 10 health priorities identified in the I-Priorities Study across Queensland: a culturally grounded approach with community perspectives, 2025

Table 1 presents a heatmap of the ranking of community-identified health priorities across the study sites in Queensland. The most frequently cited priority was “*Awareness and access to health services*”, which emerged as the top health priority overall (Table 1; Fig. 4). In contrast, lower-ranked priorities tended to be more site-specific. Notably, these less frequently mentioned themes often overlapped with broader health themes, specifically those in the top ten. For example, while “*Nutrition*” was identified as a standalone theme, it was also featured prominently within discussions related to “*Children’s health and safety*”, *“Chronic diseases*” and “*Social determinants of health*”, highlighting the interconnectedness of community health priorities. Furthermore, it is important to note that the ranking of themes reflects the language used by participants and service groups. Some groups articulated priorities using broad terms such as “*Social Determinants of health*”, while others discussed these issues through specific, lived categories such as housing, financial security, cost of living and employment.

**Table 1:**
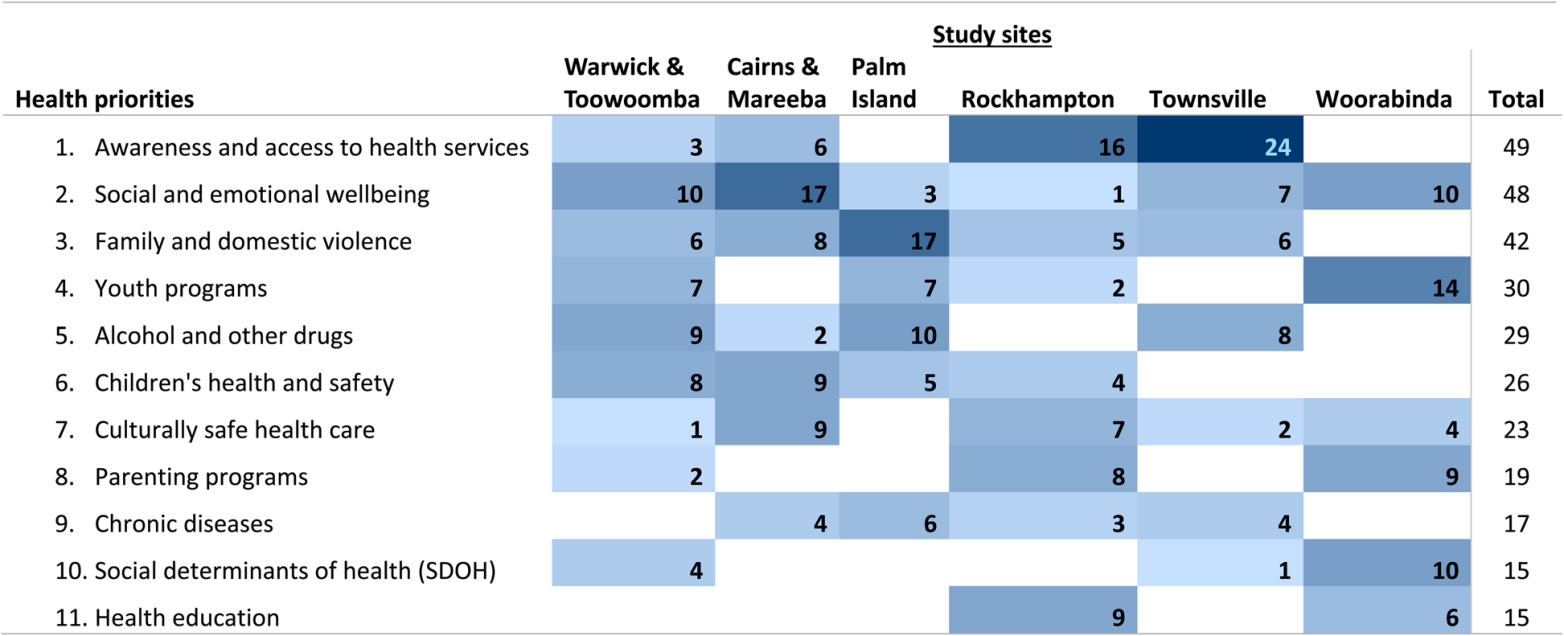
Community-identified health priorities and aggregated rankings across all I-Priorities study sites in Queensland

Numbers in rows indicate the rank of health theme at each site. *Dark blue* refers to the *highest-ranked priorities*, and *light blue* to the *lowest*. Rankings were converted to points (e.g., 1st = 10, 2nd = 9), and aggregated totals (shown in the last column) determined the overall priority order across all sites

Across the sites, there was a strong emphasis on culturally safe, community-led and holistic care. As the top priority theme, improving awareness and access to health services, particularly during pregnancy and postpartum, was greatly agreed upon. While barriers such as long wait times, financial strain and staffing shortage were noted by some participants, limited workforce capacity contributed to delays in accessing specialist care. Communities also highlighted that the underrepresentation of Indigenous health professionals constrained culturally responsive care and reduced community engagement with health systems. Despite these challenges, participants shared examples of proactive outreach and trusted relationships maintained with ACCHSs. Services such as Carbal, TAIHS and Bidgerdii were praised by study participants for their inclusive, wrap-around models that integrate health, social and cultural support. The importance of gender-specific care, especially for Women’s and Men’s business, was emphasised across regions to support culturally appropriate care based on different gendered needs. Community members consistently emphasised the importance of culturally responsive care, continuity of care and the role of trusted health workers in building engagement and trust. Participants in some regions also stressed the need for better awareness and promotion of available services, alongside improved transport and outreach programs to bridge gaps in access.

Equally prominent was the theme of social and emotional wellbeing, with mental health, grief and loss, trauma, and family and domestic violence interwoven throughout the yarning discussions covering these topics (Fig. 4). Furthermore, trauma-informed care was highlighted by communities, including the strong desire to move beyond deficit narratives, focusing instead on strengths-based, prospective approaches that honour cultural identity and resilience. Programs that connect people to Country, culture and community, such as yarning circles, cultural camps and learning on Country playgroups, were seen as significant enablers of healing and wellbeing. Youth programs, parenting support, and early childhood interventions were identified as priorities, with many communities already leading innovative, culturally grounded initiatives. Chronic diseases such as diabetes and rheumatic heart disease were prevalent, often exacerbated by poor nutrition and limited access to healthy food options. The social determinants of health, including challenges related to housing, education, employment and systemic racism, were consistently identified as underlying factors impacting health outcomes. While “social determinants of health” did not always appear as a single, high‑ranking category for some communities, its constituent elements emerged consistently and prominently across discussions. Across all themes, communities consistently emphasised commitment to self-determination, cultural revitalisation and community-led solutions that will further improve health and wellbeing.

## Discussion

The I-Priorities study provides critical insights into the priorities Indigenous communities across Queensland hold for their health and wellbeing. The strongest message delivered by communities involved in the research was that access to health services remains a profound challenge, shaped not only by geographic and financial barriers, but also by systemic issues such as institutional racism and fragmented service delivery. This aligns with broader literature demonstrating that Indigenous peoples often face structural barriers to equitable healthcare, highlighting that health equity requires more than the availability of services, it demands acceptability, affordability and cultural safety [48, 49].

The apparent lower prioritisation of “*social determinants of health*” warrants closer interpretation. Rather than indicating reduced importance, this reflects how participants articulated priorities through concrete, lived experiences such as housing insecurity, financial stress, cost of living pressures and employment challenges, rather than through abstract or academic terminology. In this sense, social determinants were highly salient across discussions, but were dispersed across multiple thematically specific categories rather than aggregated under a single label. As a result, their importance may appear less prominent when viewed only through ranked themes, despite being discussed frequently across communities.

The I‑Priorities study was intentionally designed to seek what Indigenous communities themselves prioritise across early life, perinatal and family health. The findings therefore provide guidance on where future Indigenous‑led research efforts should be directed, which areas of service development or redesign warrant prioritisation and which policy actions are most closely aligned with community‑articulated needs. By informing agenda‑setting before the design, implementation or evaluation of programs, the study contributes a foundation to ensure that subsequent research, service and policy initiatives are grounded in priorities defined by communities themselves. While participatory and co‑design approaches have been increasingly applied in Indigenous health research [34, 43, 50], many priority‑setting exercises are confined to specific communities, service settings or health domains. The I‑Priorities study extends this work through a large‑scale, multi‑site process across Queensland, combining culturally grounded yarning methods with a structured Delphi technique to support both local specificity and cross‑site synthesis. This enabled the identification and ranking of priorities across health, social and cultural domains, offering a level of breadth and comprehensiveness that is less commonly reported in Indigenous family and early life health research.

The I-Priorities study demonstrates that access is inseparable from the issue of cultural safety. It highlights the disengagement of Indigenous families from care when they feel unheard or culturally disrespected, a phenomenon well documented in Australian-wide evidence on racism in healthcare [6, 51–53]. Cultural safety requires health providers to critically examine power relations and institutional practices, shifting the onus away from patients adapting to systems and toward systems reforming to be safe and welcoming [54]. Queensland’s *Making Tracks Together* framework reflects this by mandating actions to eliminate racism and redesign health services in collaboration with Indigenous communities by 2031 [55]. The I-Priorities study’s findings reinforce that cultural safety [56] is not optional but essential for trust, engagement, and better health outcomes. Unlike cultural competency, which often focuses on individual knowledge, cultural safety strives for systemic change [57]. It is critical for dismantling institutional racism and advancing health equity, requiring health organisations to move beyond tokenistic measures and assume accountability to Indigenous patients and communities for the quality and safety of care delivered [57, 58].

This study highlighted the success and importance of holistic models of care, particularly those provided by ACCHS. Participants consistently praised ACCHS as delivering community-embedded, culturally responsive, and wrap-around services, which resonate with Indigenous understandings of health as a balance of physical, cultural, social and spiritual wellbeing [2, 17]. Evidence confirms that ACCHS overcome barriers by offering culturally safe, low-cost or free healthcare, and delivering programs that embed health with cultural identity and family support [20, 23]. Furthermore, a 2020 review of 67 ACCHO annual reports demonstrated that these services extend beyond clinical care to address social determinants of health, including cultural cohesion, socioeconomic empowerment, and political advocacy, underscoring their role as comprehensive primary healthcare providers [59]. A recent systematic review across Australia, Canada, the US and Aotearoa/New Zealand confirmed that the most effective integrated models are community-led, contextually tailored, and supported by strong partnerships, flexible delivery, skilled providers and adequate funding [60]. The I-Priorities findings thus strengthen the case for investment in community-controlled and holistic models as essential vehicles for improving Indigenous health outcomes in Queensland.

Another urgent priority identified was the need for representation of Indigenous health professionals in the workforce. Communities clearly articulated that Indigenous staff create an environment of safety and trust, yet the demand far outweighs the supply. This mirrors broader national policy commitments, such as the *National Aboriginal and Torres Strait Islander Health Workforce Strategic Framework and Implementation Plan 2021–2031*, which calls for proportional representation across the health system [61]. Indigenous practitioners bring lived experience, cultural knowledge, and language that enhance both engagement and quality of care [62]. However, systemic changes are needed to recruit, retain and support this workforce so they are not joining culturally unsafe organisations or being overburdened or isolated within government health services.

The implications of these findings are significant. For research, Indigenous-led methods including yarning and PAR must continue to guide research priorities, ensuring alignment with community needs and aspirations [24, 37]. For service design, co-design with Indigenous stakeholders, formal partnerships with ACCHS and embedding wrap-around services are necessary. For policy, alignment with frameworks such as the Federal *Closing the Gap *[63] and Queensland State *Making Tracks Together* provides an opportunity to operationalise these priorities. Concrete actions include subsidising transport and accommodation for families, mandating anti-racism training across health services, and scaling up workforce development to strengthen Indigenous leadership and cultural capability within maternity and child health care. This includes expanding initiatives such as Birthing on Country, which integrate clinical care with cultural practices and community governance to deliver safer, more empowering maternity experiences for Indigenous women [64].

A major strength of this study was the strong leadership and active involvement of Indigenous community members across all stages of the research, ensuring that diverse perspectives shaped the design, implementation and interpretation of findings. The study is unique in the Australian context due to its state‑wide scope and the use of culturally grounded participatory methods. Furthermore, extensive engagement with partner organisations and health services fostered trust and established enduring partnerships, laying the foundation for future co-designed research initiatives, including the forthcoming birth cohort study in Queensland, the Strong Families Study. In addition, face-to-face discussions during the planning phase further strengthened relationships between community and the research team, enhancing research quality and laying the groundwork for ongoing collaboration, with several community-controlled health services expressing a strong interest in continuing to collaborate in future studies. As previously mentioned, the study produced regional community reports developed for and with local communities and ACCHS. Each report underwent community peer review prior to publication, enabling communities to verify and refine findings. Following publication, the reports were physically returned to participating communities, reinforcing the act of reciprocity [65] and ensuring findings were shared, owned and used by those who contributed to the research. Together, these processes upheld Indigenous data sovereignty and ensured that identified priorities reflected place‑based knowledge. Moreover, capacity-building opportunities were created through the identification of study champions and key staff within participating services, promoting community engagement and reinforcing the value of collaborative research. The yarning sessions also provided rich insights from both community members and health professionals, deepening understanding of health priorities and strategies to address key concerns for families. Finally, the population-based data reflected in this study aligns with broader National data [66–68] except for income levels [69].

The current study also makes an important contribution by demonstrating that while broad health priorities are shared across Aboriginal and Torres Strait Islander communities, their expression and relative importance are highly context-specific at the local level. There is clear diversity in how priorities are ranked and articulated across sites, reflecting differences in geography, service availability, cultural context and lived experience. This finding reinforces a critical insight that health policy and system-level frameworks while increasingly aligned with principles such as cultural safety, do not yet fully capture the nuanced and place-based priorities identified by communities themselves. Current policy approaches provide an important overarching direction; however, the current findings suggest that their implementation must be more flexible, locally responsive and community-led to be effective. A key strength of this study is therefore its ability to illuminate the diversity of need within and across communities. This strengthens the case for decentralised, co-designed approaches to health research, service delivery and policy, where communities are not only consulted but positioned as leaders in defining and responding to their own health priorities.

However, there are several limitations that should be acknowledged. First, although the project engaged 403 participants across diverse regions of Queensland, participation was shaped by the availability of local health services and community-controlled organisations, meaning that some communities and language groups were not represented. As with all qualitative and participatory research, findings reflect the experiences and priorities of those who chose to participate and may not be generalisable to all Indigenous families in Queensland. Additionally, the predominance of women in both study phases may have limited the depth of perspectives from men, young fathers, and male caregivers, particularly relating to Men’s business. This is critical because men’s perspectives are integral to Indigenous knowledge systems, shaping their roles in family life, cultural continuity, and distinct approaches to health decision-making, an importance consistently highlighted in the literature on gender-specific care among Indigenous peoples [70–72]. While the use of yarning and the Delphi method strengthened cultural safety and community validation, these approaches rely on group dynamics and may have constrained the expression of sensitive or dissenting views. Finally, logistical constraints, including variability in the timing of site visits and differing levels of staff availability, may have influenced the breadth of perspectives captured. Despite these limitations, the study provides a robust and culturally responsive foundation for understanding community-identified health priorities.

## Conclusion

The I-Priorities study demonstrates the power of Indigenous-led research in identifying community-defined health priorities across Queensland, Australia. By centring access, cultural safety, holistic models of care, and Indigenous workforce representation, the study offers both a roadmap and a mandate for systemic change. These findings confirm that health equity will not be achieved through biomedical interventions alone but requires deep investment in cultural, social, and structural determinants of health. In this study, these determinants were articulated through lived and relational concerns, such as access to culturally safe services, cost of living pressures, housing, transport and racism, and were therefore embedded within other top‑ranking priority areas. Importantly, the study highlights that Indigenous methods such as yarning and PAR can produce not only rigorous evidence but also strengthen community trust and cultural continuity. The alignment of these priorities with state and national policy frameworks, including *Making Tracks Together* and *Closing the Gap*, underscores their timeliness and legitimacy. Taken together, this work provides a compelling case for embedding Indigenous voices and leadership at every level of health and wellbeing research, service design, and policy to ensure stronger beginnings and healthier futures for Aboriginal and Torres Strait Islander families.

## Supplementary Information

Supplementary Material 1.

## Data Availability

Data are available upon reasonable request. Please contact Prof Kym Rae (kym.rae@uq.edu.au) for more information. As mentioned in the methods section in the main document, detailed findings for most individual study sites have been published in separate reports, including those for the Far North Queensland, Darling Downs, Townsville, Palm Island, and Rockhampton regions. Corresponding citations for each report are listed in the methods section.
