# Supplementary material for: The Indigenous Health Research Priorities Study: family research priorities identified by Aboriginal and Torres Strait Islander communities in Queensland, Australia
Source: BMC Public Health. 2026 Jun 8;26:2019. doi: 10.1186/s12889-026-27521-y (PMC13326354; doi:10.1186/s12889-026-27521-y)
Supplement: Supplementary file 1 — Supplementary Material 1. [file 12889_2026_27521_MOESM1_ESM.docx]

**Supplementary Material**

**The Indigenous Health Research Priorities Study: priorities identified by Aboriginal and Torres Strait Islander communities in Queensland, Australia**

Kai Wheeler^+#1^, Salma M Ahmed^+2^, Luciana Massi^2^, Loretta Weatherall^# 2^, Davina Smith^# 2^, Rhiannon Friday^# 2^, Emily S. Dorey^2^, Bronwyn Fredericks^# 3^, Maree Toombs^# 4^, and Kym M. Rae^2,5^ *

*^+^Co-first Author*

*^#^ author identifies as Aboriginal and/or Torres Strait Islanders*

^1^ School of Human Movement and Nutrition Sciences, The University of Queensland, St Lucia, QLD, Australia

^2^ Indigenous Health Research Group, Mater Research Institute- The University of Queensland, South Brisbane, Australia

^3^ Office of the Deputy Vice Chancellor Indigenous Engagement, The University of Queensland, St Lucia, QLD, Australia

^4^ School of Population Health, Faculty of Medicine and Health, University of New South Wales, Sydney, NSW, Australia

^5^ Faculty of Health Medicine and Behavioural Sciences, The University of Queensland, St Lucia, QLD, Australia

**Corresponding author*

Professor Kym M. Rae

Address: Mater Research Institute - UQ,

Level 3, Aubigny Place,

South Brisbane, QLD, 4101

AUSTRALIA

Email: [kym.rae@uq.edu.au](mailto:kym.rae@uq.edu.au)

# **Supplementary Table 1:** Summary of engagement activities and participant numbers by site in the I-Priorities Study

| Site | Dates | Engagement Type | Participant Type | N |
| --- | --- | --- | --- | --- |
| North Queensland |  |  |  |  |
| Queensland Health (Townsville) | 11/09/2023 | Yarning | Staff | 8 |
|  | 14/09/2023 | Yarning | Staff | 5 |
| Townsville Aboriginal and Islander Health Service | 31/07/2023 | Yarning | Staff | 4 |
|  | 01/08/2023 | Yarning | Community | 2 |
|  | 01/08/2023 | Yarning | Staff | 10 |
|  | 02/08/2023 | Yarning | Staff and Community | 4 |
|  | 13/09/2023 | Yarning | Community | 8 |
|  | 14/09/2023 | Yarning | Staff | 17 |
|  | 17/10/2023 | Yarning | Community | 6 |
|  | 21/11/2023 | Delphi | Staff | 15 |
|  | 21/11/2023 | Delphi | Staff | 16 |
| Palm Island Community Company | 19/09/2023 | Yarning | Community | 4 |
|  | 19/09/2023 | Yarning | Staff | 4 |
|  | 20/09/2023 | Yarning | Community | 5 |
|  | 20/09/2023 | Yarning | Community | 3 |
|  | 21/09/2023 | Yarning | Community | 2 |
|  | 22/11/2023 | Delphi | Staff and Community | 6 |
| Central Queensland |  |  |  |  |
| Yoonthalla Services Woorabinda | 10/10/2023 | Yarning | Staff | 6 |
|  | 25/10/2023 | Yarning | Staff | 7 |
|  | 25/10/2023 | Yarning | Community | 2 |
|  | 31/01/2024 | Yarning | Staff and Community | 4 |
|  | 29/02/2024 | Yarning | Community | 14 |
|  | 23/10/2024 | Delphi | Community | 12 |
| Queensland Health (Rockhampton) | 11/10/2023 | Yarning | Staff | 11 |
|  | 27/10/2023 | Yarning | Staff and Community | 3 |
|  | 29/01/2024 | Delphi | Staff | 7 |
| Rockhampton Community Group | 12/10/2023 | Yarning | Community | 10 |
| Mount Morgan Community Group | 21/10/2023 | Yarning | Community | 12 |
| Bidgerdii Community Health Service (Rockhampton) | 24/10/2023 | Yarning | Community | 6 |
|  | 26/10/2023 | Yarning | Community | 7 |
|  | 27/10/2023 | Yarning | Staff | 15 |
|  | 30/01/2024 | Delphi | Community | 9 |
|  | 30/01/2024 | Delphi | Staff | 10 |
| Queensland Health (Emerald) | 09/10/2023 | Yarning | Staff | 5 |
| Far North Queensland |  |  |  |  |
| Wuchopperen Health Service (Cairns) | 18/05/2022 | Yarning | Staff | 5 |
|  | 08/08/2022 | Yarning | Staff | 7 |
|  | 09/08/2022 | Yarning | Community | 2 |
|  | 07/12/2022 | Delphi | Staff and Community | 14 |
| Mookai Rosie Bi-Bayan (Cairns) | 15/05/2022 | Yarning | Staff | 6 |
|  | 18/05/2022 | Yarning | Community | 8 |
|  | 09/08/2022 | Yarning | Staff and Community | 3 |
|  | 08/12/2022 | Delphi | Staff and Community | 10 |
| Mulungu Aboriginal Corporation Primary Healthcare Service (Mareeba) | 16/05/2022 | Yarning | Staff | 9 |
|  | 17/05/2022 | Yarning | Staff | 8 |
|  | 17/05/2022 | Yarning | Community | 13 |
|  | 27/02/2023 | Delphi | Staff and Community | 12 |
| Darling Downs |  |  |  |  |
| Carbal Aboriginal Medical Service (Warwick) | 02/02/2023 | Yarning | Staff | 5 |
|  | 05/05/2022 | Delphi | Staff | 5 |
|  | 28/10/2022 | Yarning | Community | 9 |
|  | 05/05/2022 | Delphi | Community | 3 |
| Carbal Aboriginal Medical Service (Toowoomba) | 20/02/2023 | Yarning | Community | 7 |
|  | 20/10/2022 | Yarning | Staff | 10 |
|  | 19/05/2023 | Delphi | Staff | 8 |

# **Supplementary Table 2** Demographic characteristics of the I-Priorities study sites in Queensland

| Demographic characteristics | Phase 1  (Yarning) | | Phase 2  (Delphi) | | Total |
| --- | --- | --- | --- | --- | --- |
|  | n = 276 | | n = 127 | | n = 403 |
|  | n | % | n | % | n (%) |
| Age range (years): |  |  |  |  |  |
| <20 | 3 | 1.1 | 0 | 0.0 | 3 (0.8) |
| 20 - 29 | 39 | 14.9 | 16 | 13.1 | 55 (14.3) |
| 30 - 39 | 57 | 21.8 | 30 | 24.6 | 87 (22.7) |
| 40 - 49 | 51 | 19.5 | 27 | 22.1 | 78 (20.3) |
| 50 - 59 | 58 | 22.1 | 28 | 23.0 | 86 (22.4) |
| 60 - 69 | 39 | 14.9 | 17 | 13.9 | 56 (14.6) |
| 70 - 79 | 11 | 4.2 | 4 | 3.3 | 15 (3.9) |
| 80 - 89 | 4 | 1.5 | 0 | 0.0 | 4 (1.0) |
| Total | **262** |  | **122** |  | **384** |
| *Missing* | *14* |  | *5* |  | *19* |
| Identity |  |  |  |  |  |
| Aboriginal | 172 | 64.2 | 80 | 63.5 | 252 (64.0) |
| Torres Strait Islander | 15 | 5.6 | 10 | 7.9 | 25 (6.3) |
| Aboriginal and Torres Strait Islander | 18 | 6.7 | 22 | 17.5 | 40 (10.2) |
| Other | 63 | 23.5 | 14 | 11.1 | 77 (19.5) |
| Total | **268** |  | **126** |  | **394** |
| *Missing* | *8* |  | *1* |  | *9* |
| Gender |  |  |  |  |  |
| Female | 235 | 86.1 | 112 | 88.2 | 347 (86.8) |
| Male | 38 | 13.9 | 15 | 11.8 | 53 (13.3) |
| Total | **273** |  | **127** |  | **400** |
| *Missing* | *3* |  | *0* |  | *3* |
| Marital status |  |  |  |  |  |
| Single | 106 | 39.8 | 50 | 41.0 | 156 (40.2) |
| Married | 73 | 27.4 | 31 | 25.4 | 104 (26.8) |
| De facto | 53 | 19.9 | 29 | 23.8 | 82 (21.1) |
| Separated | 25 | 9.4 | 9 | 7.4 | 34 (8.8) |
| Divorced | 2 | 0.8 | 2 | 1.6 | 4 (1.0) |
| Widowed | 7 | 2.6 | 1 | 0.8 | 8 (2.1) |
| Total | **266** |  | **122** |  | **388** |
| *Missing* | *10* |  | *5* |  | *15* |
| Number of children: |  |  |  |  |  |
| Nil | 50 | 18.1 | 25 | 19.7 | 75 (18.6) |
| 1 - 2 | 88 | 31.9 | 37 | 29.1 | 125 (31.0) |
| 3 - 5 | 111 | 40.2 | 58 | 45.7 | 169 (41.9) |
| > 5 | 27 | 9.8 | 7 | 5.5 | 34 (8.4) |
| Total | **276** |  | **127** |  | **403** |
| *Missing* | *0* |  | *0* |  | *0* |
| Number of grandchildren: |  |  |  |  |  |
| Nil | 182 | 66.7 | 72 | 58.5 | 258 (64.2) |
| 1 - 2 | 31 | 11.4 | 22 | 17.9 | 55 (13.7) |
| 3 - 5 | 19 | 7.0 | 12 | 9.8 | 31 (7.7) |
| > 5 | 41 | 15.0 | 17 | 13.8 | 58 (14.4) |
| Total | **273** |  | **129** |  | **402** |
| *Missing* | *3* |  | *4* |  | *3* |
| Number of dependents who are not biological children: |  |  |  |  |  |
| Nil | 231 | 83.7 | 106 | 83.5 | 337 (83.6) |
| 1 - 2 | 30 | 10.9 | 13 | 10.2 | 43 (10.7) |
| 3 - 5 | 13 | 4.7 | 6 | 4.7 | 19 (4.7) |
| > 5 | 2 | 0.7 | 2 | 1.6 | 4 (1.0) |
| Total | **276** |  | **127** |  | **403** |
| *Missing* | *0* |  | *0* |  | *0* |
| Education: |  |  |  |  |  |
| Less than high school | 51 | 19.2 | 14 | 11.1 | 65 (16.6) |
| Completed high school | 53 | 20.0 | 36 | 28.6 | 89 (22.8) |
| Some post-school education (TAFE, apprenticeship, university) | 161 | 60.8 | 76 | 60.3 | 237 (60.6) |
| Total | **265** |  | **126** |  | **391** |
| *Missing* | *11* |  | *1* |  | *12* |
| Employment: |  |  |  |  |  |
| Unemployed | 28 | 10.3 | 13 | 10.4 | 41 (10.4) |
| Employed | 182 | 67.2 | 95 | 76.0 | 277 (69.9) |
| Stay at home parent | 34 | 12.5 | 10 | 8.0 | 44 (11.1) |
| Retired | 10 | 3.7 | 5 | 4.0 | 15 (3.8) |
| Disability | 17 | 6.3 | 2 | 1.6 | 19 (4.8) |
| Total | **271** |  | **125** |  | **396** |
| *Missing* | *5* |  | *2* |  | *7* |
| Role/s in community: |  |  |  |  |  |
| Community members | 73 | 28.6 | 28 | 24.1 | 101 (27.2) |
| Health care professional | 75 | 29.4 | 40 | 34.5 | 115 (31.0) |
| Worker in organisations | 62 | 24.3 | 42 | 36.2 | 104 (28.0) |
| Worker in policy and practice | 17 | 6.7 | 0 | 0.0 | 17 (4.6) |
| Other | 28 | 11.0 | 6 | 5.2 | 34 (9.2) |
| Total | **255** |  | **116** |  | **371** |
| *Missing* | *21* |  | *11* |  | *32* |
| Household income (annual): |  |  |  |  |  |
| 0 | 3 | 1.1 | 0 | 0.0 | 3 (0.8) |
| < $28,050 (< $561) | 25 | 9.4 | 7 | 5.8 | 32 (8.3) |
| $28,051 - $49,900 ($562-$998) | 34 | 12.8 | 14 | 11.7 | 48 (12.5) |
| $49,901 - $81,450 ($999-$1,629) | 57 | 21.5 | 36 | 30.0 | 93 (24.2) |
| $81,451 - $124,600 ($1,630-$2,492) | 50 | 18.9 | 24 | 20.0 | 74 (19.2) |
| ≥ 124,601 (≥ $2,493) | 12 | 4.5 | 1 | 0.8 | 13 (3.4) |
| Social assistance | 9 | 3.4 | 3 | 2.5 | 12 (3.1) |
| Disability pension | 14 | 5.3 | 10 | 8.3 | 24 (6.2) |
| Don't know | 20 | 7.5 | 9 | 7.5 | 29 (7.5) |
| Prefer not to say | 41 | 15.5 | 16 | 13.3 | 57 (14.8) |
| Total | **265** |  | **120** |  | **385** |
| *Missing* | *11* |  | *7* |  | *18* |
| Do you have a regular healthcare provider?: |  |  |  |  |  |
| GP | 225 | 87.2 | 106 | 91.4 | 331 (88.5) |
| None | 12 | 4.7 | 3 | 2.6 | 15 (4.0) |
| Other | 21 | 8.1 | 7 | 6.0 | 28 (7.5) |
| Total | **258** |  | **116** |  | **374** |
| *Missing* | *18* |  | *11* |  | *29* |
